# Evaluation of the Antimicrobial Effect of the Extracts of the Pods of *Piliostigma thonningii* (Schumach.) Milne-Redh. (Fabaceae)

**DOI:** 10.1155/2021/6616133

**Published:** 2021-02-13

**Authors:** Memory Makosa, Simbarashe Sithole, Stanley Mukanganyama

**Affiliations:** Department of Biochemistry, University of Zimbabwe, Mt. Pleasant, Harare, Zimbabwe

## Abstract

Plants have been used traditionally by people in treating and the management of diseases since time immemorial. Traditional medicines including the herbal medicines are used for primary healthcare in some domains in almost every country. Approximately 80% of the population in developing coutries depend on plants as their source of medicine for combating diseases. New and effective antimicrobial agents that have novel mechanism of actions are required. *Piliostigma thonningii* (Schumach.) Milne-Redh. is a species of flowering plants in the legume family, Fabaceae. Different parts of the *P. thonningii* plants such as the roots, leaves, seeds, and fruits have been used in treating wounds, heart pain, and gingivitis and as cough remedy. This study focused on determining the antimicrobial properties found in the pods of *P. thonningii*. The sample was prepared by grinding the dried pods into a fine powder. Successive extraction and extraction with 1 : 1 DCM: methanol was used. The antimicrobial assay was carried out using the broth microdilution and MTT assay. The microorganism used for the tests was *Pseudomonas aeruginosa*, *Candida krusei* and *Mycobacterium smegmatis*. The most potent extract was then used to determine its effect on microbial cell membrane integrity. The results showed that methanol extract had the highest percentage yield of 5%. The extract with the highest antimicrobial effects was ethanol extract with the 100 *μ*g/mL concentration inhibiting the growth of cells to 26%, 87%, and 90% for *P. aeruginosa*, *M. smegmatis,* and *C. krusei,* respectively. The ethanol extracts caused significant leakage of proteins in these microorganisms. In conclusion, the pods of *P. thonningii* contain phytochemicals with antimicrobial properties. The pods of the plant can be a source of phytochemicals that can serve as sources of lead compounds with antimicrobial effects. One of the mechanisms of action of these phytochemicals is via membrane-damaging effects on microbes.

## 1. Introduction

Plants have long been used in the prevention, diagnosis, and treatment of different physical as well as mental illnesses. About 50% of current drugs are derived either directly or indirectly from plants, with approximately 80% of the population in developing countries relying exclusively on plants as sources of medicines [1]. Several studies have shown that medicinal plants are composed of bioactive compounds with proven efficacy as the basic raw material of drugs [2]. In Zimbabwe, there are more than 5000 species of plants of which only 10% of themhave been used for medicinal properties. Traditional medicine is still regarded as the cheapest and most accessible source of treatment against diseases in many communities in Zimbabwe [3].

An antimicrobial agent is defined as the agent that inhibits the growth or destroy microorganism [4]. Secondary metabolites have contributed to the antimicrobial potency of natural products against several diseases. The therapeutic effects of medicinal plants showed physiological action on the human body. Hence, people use them for the general maintenance of health [5]. Medicinal plants have frequently been used as raw materials for the extraction of bioactive constituents for the production of different drugs [6]. In a study to explore and record plants used by the traditional healers of South Africa, *P. thonningii* roots, bark, and leaves were found to be used to treat loss of appetite, or for alleviating stomach problems as well as haematochezia [7].

*Piliostigma thonningii Schum.* is a leguminous plant that belongs to the family Fabaceae and Caesalpinioideae subfamily which comprises 133 genera [8]. The crude extract of *P. thonningii* has been reported to possess antilipidemic, antibacterial, antihelminthic, and anti-inflammatory activities [2]. The presence of bioactive compounds such as flavonoids, tannins, kaurane diterpenes, alkaloids, carbohydrates, saponins, terpenes, and volatile oils has shown the potential of inhibiting the growth of pathogens [9]. Most of the previous studies on the plant *P. thonningii* were on leaves, with these studies demonstrating anti-inflammatory, analgesic, and toxic effects of the leaf extracts [10]. In a recent study, it was shown that aqueous, and methanolic stem bark extracts of *P. thonningii*, possessed analgesic and anti-inflammatory activities [11]. Antifungal screening on the aqueous and ethanolic extracts of *P. thonningii* leaves has shown them to have antifungal effects on *Colletotrichum musae* and *Sclerotium rolfsii* [12]. *P. thonningii* stem bark 60% methanolic extract has been reported to possess antibacterial activity against *Bacillus subtilis*, *Corynebacterium pyogenes*, *Escherichia coli*, *Proteus vulgaris*, *Shigella dysenteriae,* and *Staphylococcus aureus* [13].

Considerable information exists on the phytochemicals present in *P. thonningii*. In an investigation by Ighodaro et al. [14], they found that the aqueous and ethanol leaf extracts of the plant contained alkaloids, saponins, flavonoids, and tannins. In the same study, the extracts when tested for their antibacterial as well as antifungal activity showed effective inhibition of growth of human pathogenic microbes [14]. Moriasi et al. [15] qualitatively screened for the phytochemicals of the aqueous and methanolic stem bark extracts of *P. thonningii* and observed the presence of cardenolide glycosides, coumarins, phenols, steroids, saponins, and flavonoids.

*Candida albicans, Candida glabrata, Candida tropicalis, Candida parapsilosis,* and *Candida krusei* are five *Candida* species that cause 92% of cases of candidemia [16]. *Mycobacterium smegmatis* was discovered firstly to be a human pathogen in 1986 by Vonmoos [17]. From then, many cases of mycobacterial infections have been reported of which about 56–76% are of skin or soft-tissue infections. *P. aeruginosa*causes nosocomial infection in hospitalised patients and community-acquired infections. *P. aeruginosa* has been reported to be the second organism amongst others causing ventilator-associated pneumonia, catheter-associated urinary tract infections, wound infections in severe burn patients, and septicaemia [18]. In many developing as well as developed countries, there is indiscriminate use of antimicrobials that has led to the development of microbial resistance problems [19]. The rise of antimicrobial resistance had led to high cost, long period, and failure of treatment often leading to the death of patients. In an effort to stem the cost of producing as well as the comparatively high adverse effects of synthetic drugs, studies are being undertaken into the search for new antimicrobial substances from natural sources [20].

The pods of *P. thonningii* have not been investigated the antimicrobial potency, unlike other different parts which include leaves, stem, and roots. This study focused on determination of the antimicrobial properties found in the pods of *P. thonningii*. The extracts of the pods are tested against three types of microbial cells: *P. aeruginosa*, *C. krusei,* and *M. smegmatis* to determine the antimicrobial potency.

## 2. Materials

### 2.1. Chemicals and Reagents

All the chemicals and reagents used were obtained from Sigma-Aldrich Chemical Co. (Darmstadt, Germany). All solvents used were of analytical reagent grade; these were acetone, methanol, n-hexane, ethanol, dichloromethane, 3-(4,5-dimethylthiazolyl)-2,5-diphenyltetrazolium bromide (MTT), potassium hydroxide (KOH), and dimethyl sulfoxide (DMSO). The pods of *Piliostigma thonningii* were obtained from Norton, a town located in Mashonaland West Province of Zimbabwe (17°53′0″ south, 30°42′0″ east). The pods were identified and authenticated at the National Herbarium and Botanical Gardens at Harare by a taxonomist Mr. Christopher Chapano.

### 2.2. Bacterial Strain and Plant Material

The clinical strain of *P. aeruginosa* used during the tests was acquired from Parirenyatwa Hospital (Department of Medical Microbiology, College of Health Sciences, Harare, Zimbabwe). *M. smegmatis* 155 mc^2^ laboratory strain was obtained from the Department of Clinical Laboratory Sciences, University of Cape Town. *C. krusei* was obtained from the Department of Biological Science at the University of Botswana.

### 2.3. Extraction of Phytochemicals with Differential Solvents

The pods of *P. thonningii* were dried for 3 days at 40°C in an oven. The dried pods were ground using pestle and mortar until it was a fine-powdered. The powder of the pods was sieved to remove unwanted fragments. The powder was placed in a container and stored at room temperature conditions. Two methods used for extraction were total extraction and serial exhaustive extraction. A mixture of solvents, methanol and dichloromethane (1 : 1), was used in the total extraction. A 50-gram mass of the powder was measured and placed in a 1-litre beaker followed by addition of 200 mL of the solvent. The mixture was left for overnight to allow for the extraction of phytochemicals. The same procedure was also carried out on serial exhaustive extraction. The solvents used were of varied polarity and ranged from nonpolar to polar. The order of the solvents was hexane, dichloromethane, acetone, ethyl acetate, ethanol, methanol, and water. After an overnight period, the extracts were filtered using cotton in the first round to remove debris. It was followed by the use of Whatman No. 1 filter paper for further filtration. The solvents were then dried in 50 mL centrifuge tubes on a fan until they are dried. The dried extracts were measured to obtain their mass. Eight extracts were obtained and further used for the antimicrobial assay.

### 2.4. Screening for Antimicrobial Activity

All the microorganisms were grown from solid into the broth. The cells were cultured by aliquoting 20 *µ*L of media of the organism under study into 50 mL centrifuge tubes and inoculating the media with microbe inoculum. The cells were standardized using a 0.5 McFarland standard. The cells were then diluted to 2 × 10^6^ CFU/mL. The extracts were dissolved by measuring 0.004 g in 1 mL of DMSO and 0.002 g of the drug was dissolved in 1 mL DMSO. This was followed by serial dilutions which started from the highest concentration to the lowest concentration. For the highest concentration, 9.5 mL of media was added to 0.5 mL of the extracts and only 2.5 mL was transferred out from every tube upon dilutions. The concentration prepared were 6.3 *μ*g/mL, 12.5 *µ*g/mL, 25 *µ*g/mL, 50 *µ*g/mL, and 100 *µ*g/mL. The procedure was carried out on all the solvent extracts which include hexane, acetone, DCM, ethyl acetate, ethanol, methanol, water, and total extract of DCM:Methanol (1:1) extract. Standard antimicrobial agents were prepared in a DMSO: media (0.5:9.5 mL) solution. This was done for the rifampicin, ciprofloxacin, and miconazole for the positive control. After serial dilutions, the solutions were plated on the 96-well plate. After 20-hour incubation at 37°C in an incubator (Lab Companion IS 300, Jeio Tech, Korea) of microorganisms and test samples, the cell viability was determined using MTT solution. In each well, 20 *µ*L of the solution was dispensed into the well. The plate was further incubated for 2 hours at 37°C in the incubator and the density of viable cells was determined spectrophotometrically at 590 nm in a Genios Pro microplate reader (Tecan Group Ltd., Grodig, Austria).

### 2.5. Determination of the Effects of Extracts on Membrane Integrity

In order to determine the effect of *P. thonningii* extracts on the bacterial and fungal membranes, the method of Fouda et al. [21] was used. The method determines the amount of proteins that leak from the organisms after exposure to membrane permeabilizing agents. The protein leakage assay was carried out by growing the microorganism under the test samples at 50 *µ*g/mL, 100 *µ*g/mL, and 200 *µ*g/mL. Five tubes were labelled according to the respective concentration of the extract and the controls. This same procedure was carried out for all the three types of microorganisms used in this study. For positive control of *C. krusei*, miconazole was used. For *M. smegmatis*, 0.1% SDS was used. Lastly, for *P. aeruginosa*, ampicillin was used. For the 50 *µ*g/mL test sample tube, the following constituents were added, 750 *µ*L of ethanol extract and 5250 *µ*L of a cell. For 100 *µ*g/mL, 1500 *µ*L ethanol extract and 4500 *µ*L cells were added. Lastly, for 200 *µ*g/mL of ethanol extract, there was 2000 *µ*L of extract and 4000 *µ*L of the cells was added. The test with the positive control contained 600 *µ*L of a test sample and 5400 *µ*L of media. The negative control tube had 6000 *µ*L of cells in media. All the five tubes were placed for 2 hours at 37°C in an incubator (Lab Companion IS 300, Jeio Tech, Korea). 

### 2.6. Statistical Analysis

Statistical analysis was done using the one-way analysis of variance (ANOVA) test. Further analysis of the results was carried out along with Dunnett's multiple comparison posttest to compare all mean values to the mean value of a control column for each test. Values with *p* values < 0.05 at 95% confidence intervals were considered to be statistically significant. GraphPad Prism 6® (Version 6.0, GraphPad Software Inc, San Diego, United States of America) software was used for all graphical and statistical analyses.

## 3. Results and Discussion

Microorganisms such as *P. aeruginosa*, *M. smegmatis,* and *C. krusei* have been shown to the cause of nosocomial infections which are acquired in a hospital during the periods of staying. An example of infection is candidemia caused by *C. krusei* which have shown an increasing incidence in nosocomial infections [22]. *C. krusei* has become resistant to fluconazole. The mortality ranges from infections by these organisms range between 36% and 63% [23]. *P. aeruginosa*, *M. smegmatis*, and *C. krusei* have displayed a mechanism to resist the available antimicrobial agents. This has led to the search for new and effective alternative medicines that can reduce these effects imposed by these pathogens. Traditionally, *P. thonningii* has been used for the treatment of ailments, which include bilharzia, wounds, and bleeding wounds [2]. This study focused on investigating the antimicrobial effects of the extracts of *P. thonningii* pods to establish the scientific basis for the traditional uses of the plant for treating microbial infections.

### 3.1. Antimicrobial Effects of the Extracts

The extracts of the pods of *P. thonningii* against *P. aeruginosa, M. smegmatis,* and *C. krusei*were shown to be effective and comparable to the antimicrobial effects of the reference drugs. The more polar solvents were shown to be effective against all the three human pathogenic microbes.

#### 3.1.1. The Antimycobacterial Effects of the Extracts against *M. smegmatis*

**Figure 1:**
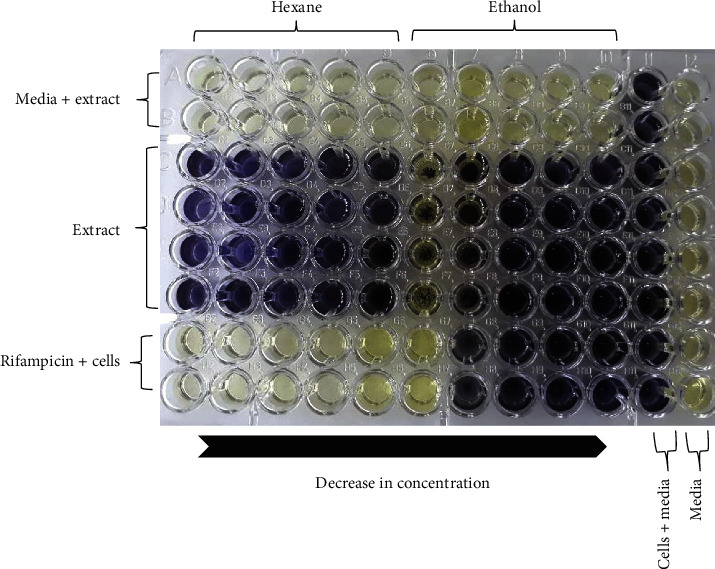
A typical microbroth dilution assay showing MTT results after subculture of *M. smegmatis* with control drug rifampicin and hexane and ethanol pod extracts of *P. thonningii.* Viable *M. smegmatis* cells reduced yellow MTT dye to blue. This indicated that there was no inhibition of *M. smegmatis* in wells with blue colour. Column 11 represents cells only well (positive control). Column 12 containing media only remained yellow. Thus, no contamination of media occurred. The top two rows A and B remained yellow, where extract and media were checked for contamination.

**Figure 2:**
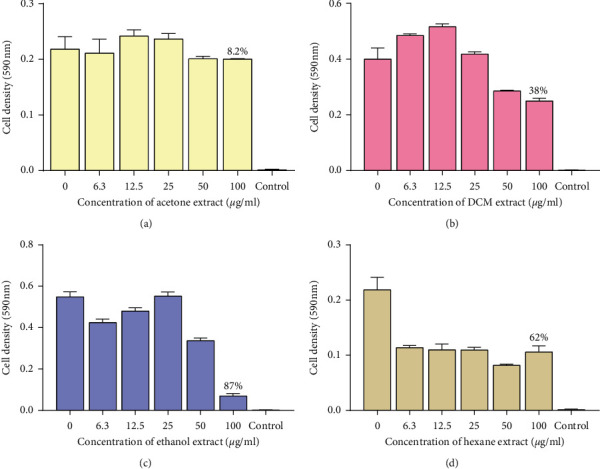
The effects of extracts of pods of *P. thonningii* against *M. smegmatis*: (a) effect of acetone extract, (b) effect of DCM extract, (c) effect of ethanol extract, and (d) effect of hexane extract. Concentrations of extract ranged from 6.3 *μ*g/mL to 100 *μ*g/mL. Values are in mean cell density at 590 nm wavelength ± the standard deviation; *N* = 4.

**Figure 3:**
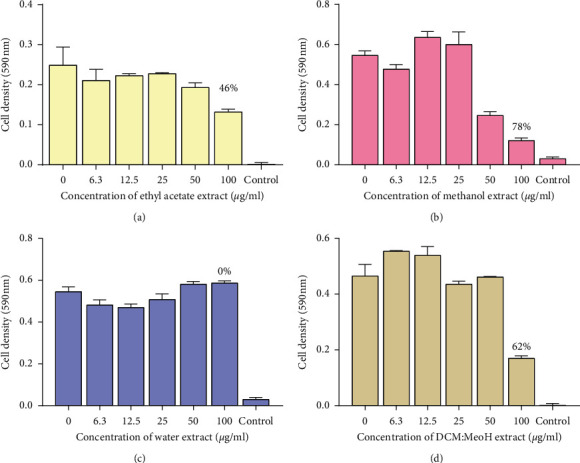
The effects of extracts of pods of *P. thonningii* against *M. smegmatis*: (a) effect of ethyl acetate extract, (b) effect of methanol extract, (c) effect of water extract, and (d) effect of DCM : MeOH extract. Concentrations of extract ranged from 6.3 *μ*g/mL to 100 *μ*g/mL. Values are in mean cell density at 590 nm wavelength ± the standard deviation; *N* = 4.

A total of eight extracts were tested against *M. smegmatis* to determine the antimycobacterial properties found in the pods of *P. thonningii*. A typical profile of the results obtained for the microbroth dilution assay for *M. smegmatis* is shown in Figure 1. The results were plotted as graphs of cell density at 590 nm against extract concentration as shown in Figures 2 and 3. Whilst there was a reduction in the growth as shown by the hexane and methanol extracts, the other extracts were infective in reducing the growth of *M. smegmatis*. No MIC was observed for all the 8 extracts against *M. smegmatis*. The ethanol extract (100 *μ*g/mL) had the highest activity with a percentage growth inhibition of 87% and followed by the methanol extract (100 *μ*g/mL) which had a percentage growth inhibition of 78%. The water extract showed no activity (Figure 3).

*M. smegmatis* has been used as a model organism for the analysis of drugs used for treating tuberculosis. *M. smegmatis* shared about 2000 homology genes with *M. tuberculosis* [24]. Tuberculosis has greatly caused high death rate around the world and it was estimated that one-third of the world population is affected by the disease [25]. The ethanol extracts from *P. thonningii* were shown to be bacteriostatic but not bactericidal. The lack of bactericidal effects can be due to the presence of cell wall made up of mycolic acids that leads to decreased antibiotic uptake renderingthe entry of drugs difficult and leading to an increase in antibacterial resistance of TB [26]. The other extracts were shown to be less effective and these were the acetone, ethyl acetate, dichloromethane, and water extracts. Comparing with a study done by Mautsa and Mukanganyama [27], the plant *Vernonia adoensis* was shown to be effective against *M. smegmatis* using extracts from the leaves and flowers. It has also been reported that the leaves of *P. thonningii* have no antitubercular activity but show remarkable general antimicrobial activity against both Gram-positive and Gram-negative bacteria [28].

#### 3.1.2. The Antibacterial Effects of the Extracts against *P. aeruginosa*

**Figure 4:**
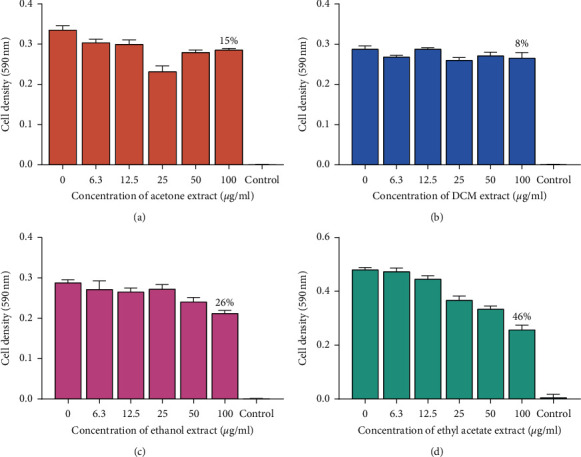
The effects of extracts of pods of *P. thonningii* against *P. aeruginosa*: (a) effect of acetone extract, (b) effect of DCM extract, (c) effect of ethanol extract, and (d) effect of ethyl acetate extract. Concentrations of extract ranged from 6.3 *μ*g/mL to 100 *μ*g/mL. Values are in mean cell density at 590 nm wavelength ± the standard deviation; *N* = 4.

**Figure 5:**
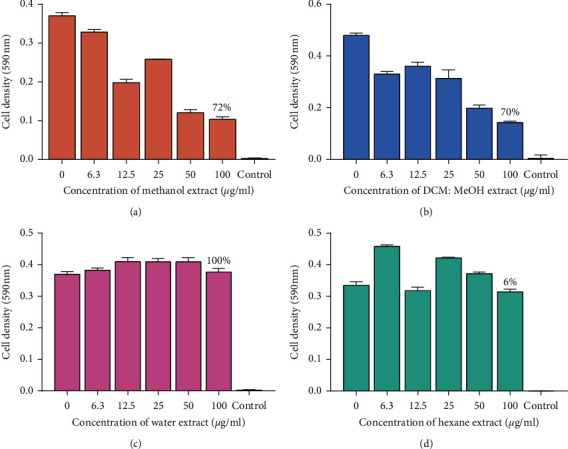
The effects of extracts of pods of *P. thonningii* against *P. aeruginosa*: (a) effect of methanol extract, (b) effect of DCM : MeOH extract, (c) effect of water extract, and (d) effect of hexane extract. Concentrations of extract ranged from 6.3 *μ*g/mL to 100 *μ*g/mL. Values are in mean cell density at 590 nm wavelength ± the standard deviation. *N* = 4.

The antibacterial effects assay of the 8 extracts against *P. aeruginosa* were determined. The results were plotted as graphs of cell density at 590 nm wavelength against the concentration of the extract shown in Figures 4 and 5. There was no MIC observed from all the 8 extracts. The methanol extract (100 *μ*g/mL) inhibited the growth of *P. aeruginosa* with a percentage inhibition of 72% followed by the exhaustive extract (methanol and dichloromethane 1 : 1 mixtures) which had a percentage inhibition of 70% at 100 *μ*g/mL of the concentration of the extracts. Other extracts at 100 *μ*g/mL, namely, hexane, acetone, ethyl acetate, ethanol, and dichloromethane, were less effective in inhibiting the growth of *P. aeruginosa*. This was attributed to the fact that Gram-negative bacteria possess an additional outer membrane composed of hydrophilic lipopolysaccharide layer that mimics the entry of hydrophobic and amphipathic compound which encompasses many drug compounds [18].

According to the study by Njeru et al. [28], the methanol crude extracts of *P. thonningii* leaves were shown to be more effective against Gram-negative bacteria with a MIC of 12.5 *μ*g/mL of the extracts. A study by Nguta et al. [29] showed that the growth of *P. aeruginosa* was inhibited by the methanol extract with a zone of inhibition between 23 mm and 24 mm. A study from Akinpelu and Obuotor [2] was of interest to our investigations as it showed that the stem bark extract of *P. thonningii* exhibited no activity against *Pseudomonas aeruginosa* and *Serratia marcescens*. In this study, no MIC was observed for any of the extracts.However, previous results showed that antibacterial property of the methanol extracts from different parts of plants can exhibit highly significant variations in the pattern of inhibition [30–32] even with no observable MIC. Our findings are in agreement with those found by Mostafa et al. [32] who found that extracts of *Rumex vesicarius* L. seeds have variable effects against both Gram-positive bacteria and Gram-negative bacteria. The presence of antibacterial property of the methanol extract *P. thonningii* corresponds to the report by Jimoh and Oladiji [33] and Daniyan et al. [34] that attributed the presence of flavonoids in the seeds and stem extracts of *P. thonningii* for its antimicrobial activity. Our results are in line with those reported by Ighodaro et al. [14] on the microbial activities of *P. thonningii* against one of the most important human pathogenic bacterium. Antibacterial activity studies by Dluya et al. [35] also reported that the methanol extract from *P. thonningii* showed activity on five selected pathogenic organisms at varied concentrations of the extract. The antibacterial activities of *P. thonningii* fruit methanol extract can be attributed to the presence of metabolic toxins as well as broad spectrum antimicrobial compounds that may act against bacteria. In another study by Dluya and Dahiru [36], it was shown that the methanol stem bark extract of *P. thonningii* significantly inhibited the growth of *S. aureus*, *S. typhi,* and *P. aeruginosa*.

#### 3.1.3. The Antifungal Effects of the Extracts against *C. krusei*

**Figure 6:**
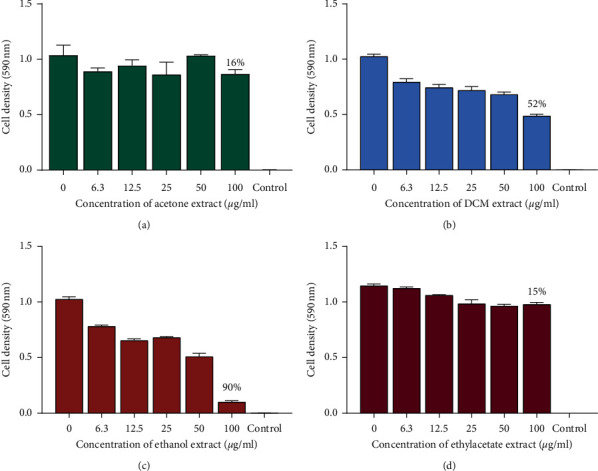
The effects of extracts of pods of *P. thonningii* against *C. krusei*: (a) effect of acetone extract, (b) effect of DCM extract, (c) effect of the ethanol extract, and (d) effect of ethyl acetate extract. Concentrations of extract ranged from 6.3 *μ*g/mL to 100 *μ*g/mL. Values are in mean cell density at 590 nm wavelength ± the standard deviation. *N* = 4.

**Figure 7:**
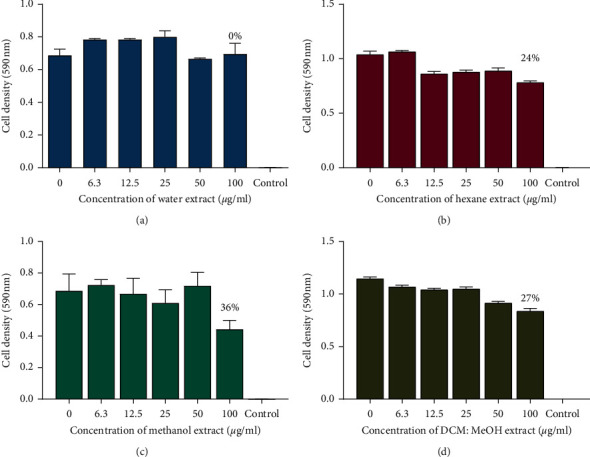
The effects of extracts of pods of *P. thonningii* against *C. krusei*: (a) effect of water extract, (b) effect of hexane extract, (c) effect of the methanol extract, and (d) effect of ethyl acetate extract. Concentrations of extract ranged from 6.3 *μ*g/mL to 100 *μ*g/mL. Values are in mean cell density at 590 nm wavelength ± the standard deviation. *N* = 4.

The antifungal assay of the 8 extracts of the pods of *P. thonningii* against *C. krusei* was determined. The results were plotted as graphs of cell density at 590 nm against the concentration of the extracts as shown in Figures 6 and 7. The results show that ethanol extract (100 *μ*g/mL) had the highest percentage growth inhibition of 90%, whilst extracts such as water showed no activity. There was no MIC observed for the concentration of all the extracts used. Other extracts at 100 *μ*g/mL showed moderate growth inhibition of 16%, 27%, 36%, and 24% for extracts acetone, DCM: MeOH, methanol, and hexane extracts, respectively.

 ofAstudy on the effect of grape seedextracts revealed that at 50 μg/ mL ofthe extract, there was inhibition of the growth of *C. krusei* [37]. The antifungal activity of *P. thonningii* against *C. albicans* was studied previously with the ethanolic extract showing significant activity in inhibiting the growth of cells and an MIC of 0.312 *μ*g/mL. Other studies [38, 39] have found that the ethanol leaf extract of *Cassia alata* caused a dose-dependent effect on *Candida albicans*, *Microsporum canis,* and *Trichophyton mentagrophyte*. The phytochemical and antifungal analysis of the ethanolic extract of *P. thonningii* showed the presence of flavonoids which were effective in inhibiting the growth of *C. albicans*, *Colletotrichum musae,* and *Sclerotium rolfsii* [40–42]. Therefore, plants and herbal compounds used in the traditional treatment of diseases including fungal infections are potential sources for the development of new antifungal agents [43]. The moderate activity of some crude extracts can be attributed to the fact that *C. krusei* has developed resistance to several drugs especially to the group of azoles that target the cell wall structure of the fungal [44]. The ability of the ethanol extract to inhibit the growth of tested organism indicates antifungal potential that may be used in the treatment of fungal infections [40].

#### 3.1.4. Effect of the Extracts on the Membrane Integrity

**Figure 8:**
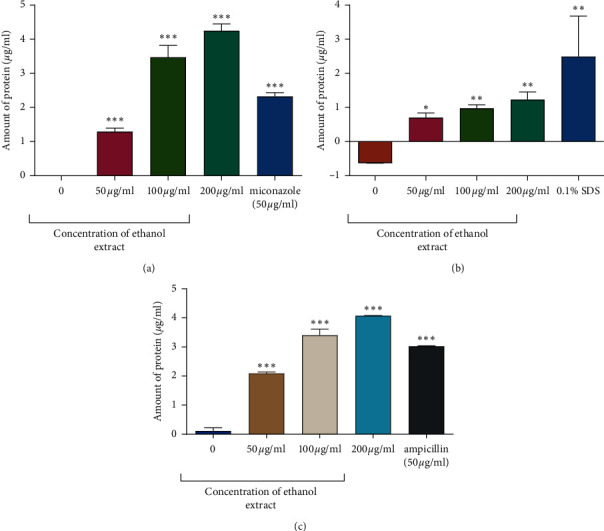
The effects of the ethanol crude on protein leakage in *C. krusei, M. smegmatis,* and *P. aeruginosa*. The results show the protein leakage assay for the effect of *P. thonningii* extract on *C. krusei* (a), *M. smegmatis* (b), and *P. aeruginosa* (c). The assay was carried out at varying concentrations of both the drug and extract. The positive control for *C. krusei* was miconazole, for *M. smegmatis* was 0.1% SDS, and for *P. aeruginosa* was ampicillin. ^*∗*^*P* < 0.05, ^*∗∗*^*P* < 0.001, and ^*∗∗∗*^*P* < 0.0001.

The most potent extracts were further used to determine the possible mode of action of the extracts by determining the effects of protein leakages from the microorganisms. The most potent extract was the ethanol extract and, thus, it was tested on all the three species. The extracts concentration used was 50 *µ*g/mL, 100 *µ*g/mL, and 200 *µ*g/mL. The positive control includes ampicillin, 0.1% SDS and miconazole for *M*. *smegmatis, C. krusei,* and *P. aeruginosa,* respectively. The protein concentration values were interpolated from the standard curve using GraphPad Prism version 5.03. The results showed that as the concentration of the ethanol extract was increased, there was significant leakage of protein from the three species (Figure 8). There was more protein leakage in *P. aeruginosa* exposed to 200 *µ*g/mL concentration of the extract giving a leakage of 4 *µ*g/mL of protein.

It was also shown that the ethanol extract caused membrane leakage in *C. krusei* and *M*. *smegmatis*. With increasing concentration of the extracts, the amount of protein that leaked also increases. In a study by Mautsa and Mukanganyama [27], exposure of *M. smegmatis* to leaf extracts from *Vernonia adoensis* caused leakage of proteins, and it was suggested that the extracts bind to the lipids and polysaccharides on the membrane leading to membrane disruption. The polar solvent extracts of six plant species have shown higher antimicrobial activity against multidrug-resistant strains of *Mycobacterium smegmatis* [45]. This has been attributed to the fact that ethanol, as the extractant, has high concentration of diterpenoids potentially contributing to the antimycobacterial activity observed in this study [46–48]. The antimycobacterial activity was carried, out and it was found that the ethanol extract affected the growth of *M. smegmatis* for *P. americana*, *Artemisia afra*, *Dodonaea angustifolia*, *Drosera capensis,* and *Galenia africana* [49–51]. These studies provide a scientific basis of our results that the ethanol extract of *P. thonningii* fruits can be used in local medicinal applications by traditional practitioners. Membrane integrity maintains pH, ATP production, and membrane potential and integrity for the physiological functions. The perturbation of the homeostasis of these biochemical molecules can form the basis action of novel antimicrobial agents [52]. Due to the development of mycobacterial persisters as well as antimycobacterial resistance, alternative treatment regimens are urgently required. The disruption of the bacterial membrane by chemical agents has emerged as an effective means to eradicate or inhibit the growth of bacteria, thereby, allowing the treatment and reduction of infectious diseases [53]. Most antifungal agents have been developed to primarily inhibit enzymes that are involved in the biosynthesis of the fungal cell membrane components such as phospholipid, sphingolipid, and ergosterol [54].

## 4. Conclusion

Antimicrobial activity was observed from extracts from the pods of *P. thonningii*. The most effective extract was ethanol extract with the 100 *μ*g/mL concentration inhibiting the growth of cells to 26%, 87%, and 90% for *P. aeruginosa*, *M. smegmatis,* and *C. krusei,* respectively. The possible mode of action of the ethanol extracts could be disruption of membrane integrity of bacteria, mycobacteria, and fungi since there was significant leakage of proteins from these microorganisms. The pods of *P. thonningii* have shown to have antimicrobial activity with membrane damaging effects on microbes. Further work on isolation and pontification of the phytochemicals from the pods is warranted.

## Data Availability

The datasets used and/or analysed during the current study are available from the corresponding author on reasonable request.
